# Assessment of Sleep Disturbances and Exhaustion in Mothers of Children With Atopic Dermatitis

**DOI:** 10.1001/jamadermatol.2018.5641

**Published:** 2019-03-20

**Authors:** Faustine D. Ramirez, Shelley Chen, Sinéad M. Langan, Aric A. Prather, Charles E. McCulloch, Sharon A. Kidd, Michael D. Cabana, Mary-Margaret Chren, Katrina Abuabara

**Affiliations:** 1 Medical student, Department of Dermatology Program for Clinical Research, University of California, San Francisco; 2 Faculty of Epidemiology and Population Health, London School of Hygiene and Tropical Medicine, London, United Kingdom; 3 Department of Psychiatry, University of California, San Francisco; 4 Department of Dermatology Program for Clinical Research, University of California, San Francisco; 5 Department of Epidemiology & Biostatistics, University of California, San Francisco; 6 Department of Pediatrics, University of California, San Francisco; 7 Philip R. Lee Institute for Health Policy Studies, University of California, San Francisco; 8 Department of Dermatology, Vanderbilt University Medical Center, Nashville, Tennessee

## Abstract

**Importance:**

The well-being and development of children is strongly influenced by parents’ physical and psychosocial health. Data from small, clinic-based studies suggest that sleep loss may be common in parents of children with atopic dermatitis (AD), but longitudinal population-based studies are lacking.

**Objectives:**

To compare sleep disturbances over time between mothers of children with and without AD and to determine whether these disturbances are associated with the child’s disease severity and the child’s sleep disturbances.

**Design, Setting, and Participants:**

In the ongoing Avon Longitudinal Study of Parents and Children, all pregnant women residing in Avon, United Kingdom, with an expected delivery date between April 1, 1991, and December 31, 1992, were recruited. Analyses for this study, a secondary analysis of this cohort, were performed from September 2017 to September 2018. Mother-child pairs were followed up with a time-varying measure of child AD activity and severity and self-reported maternal sleep measures repeated at multiple time points between child ages 6 months and 11 years.

**Main Outcomes and Measures:**

Time-varying binary measures of maternal sleep duration (<6 vs ≥6 hours per night), difficulty falling asleep, early morning awakening, subjectively insufficient sleep, and daytime exhaustion.

**Results:**

The study followed up 13 988 mother-child pairs from birth for a median duration of 11 (interquartile range, 7-11) years. Among the cohort, 11 585 of 13 972 mothers (82.9%) were aged 21 to 34 years and 12 001 of 12 324 (97.4%) were of white race/ethnicity; 7220 of 13 978 children (51.7%) were male. Sleep duration (adjusted odds ratio [AOR], 1.09; 95% CI, 0.90-1.32) and early morning awakenings (AOR, 1.16; 95% CI, 0.93-1.46) were similar between mothers of children with and without AD. In contrast, mothers of children with AD were more likely to report difficulty falling asleep (AOR, 1.36; 95% CI, 1.01-1.83), subjectively insufficient sleep (AOR, 1.43; 95% CI, 1.24-1.66), and daytime exhaustion (AOR, 1.41; 95% CI, 1.12-1.78) independent of the child’s comorbid asthma and/or allergic rhinitis. For all measures, worse child AD severity was associated with worse maternal sleep outcomes. The magnitude and significance of the associations were largely unchanged after adjustment for child sleep disturbances.

**Conclusions and Relevance:**

Mothers of children with AD reported difficulty falling asleep, subjectively insufficient sleep, and daytime exhaustion throughout the first 11 years of childhood. However, child sleep disturbances did not fully explain maternal sleep disturbances, and future research should investigate other mechanisms. In caring for children with AD, clinicians should consider maternal sleep disturbances and caregiver fatigue.

## Key Points

**Question:**

Do mothers of children with atopic dermatitis experience sleep disturbances, and to what extent are these explained by child sleep disturbances?

**Findings:**

In this cohort study of 11 649 mother-child pairs, sleep disturbances were common among mothers of children with atopic dermatitis followed up from birth through age 11 years. Having a child with atopic dermatitis was significantly associated with impaired maternal sleep quality, subjectively insufficient sleep, and increased daytime exhaustion; however, child sleep disturbances did not fully explain maternal sleep disturbances.

**Meaning:**

Clinicians caring for children with atopic dermatitis should screen for maternal sleep disturbances and caregiver fatigue.

## Teaser

This longitudinal cohort study analyzes data collected across 11 years to assess the association between the presence and severity of atopic dermatitis in children and maternal sleep disturbance among a population-based UK cohort of mother-child pairs.

## Introduction

The health, well-being, and development of children is strongly influenced by the physical and psychosocial health of parents.^1,2^ Parents of children with chronic illness, in particular, are susceptible to poor sleep, and impaired sleep has been associated with increased risks of cancer and cardiovascular disease,^3^ infectious illness,^4^ obesity,^5^ and premature mortality.^6^ Previous studies have found major sleep impairments among parents of children with ventilator dependency and cystic fibrosis,^7,8^ but few studies to date have examined sleep patterns among parents of children with more common chronic illnesses.

Atopic dermatitis (AD) is one of the most common chronic childhood conditions, affecting up to 1 in 5 children in developed countries.^9^ Atopic dermatitis is characteristically itchy, and the symptom is often reported to be more severe at night,^10,11^ causing nighttime awakenings secondary to itching and scratching.^12,13^ To our knowledge, few studies have directly examined the association between parental sleep disturbances and severity of child AD and accounted for child sleep disturbances. Existing research is limited and has focused on snapshots of small, clinic-based populations that are likely to have more severe disease than the general population.^12,13,14,15^ Because the severity and activity of AD can vary throughout childhood, longitudinal studies can provide better insight into how variations in the child’s disease activity and severity over time affect parental sleep.

We aimed to determine whether mothers of children with active AD have impaired sleep during the first 11 years of childhood and whether these sleep disturbances are associated with the child’s disease severity and the child’s sleep disturbances.

## Methods

### Participants

Data for this study come from the ongoing Avon Longitudinal Study of Parents and Children (ALSPAC), a longitudinal, population-based birth cohort in the United Kingdom.^16,17^ All pregnant women residing in Avon, United Kingdom, with an expected delivery date between April 1, 1991, and December 31, 1992, were recruited. The ALSPAC study enrolled a total of 14 541 pregnancies, which resulted in 14 062 live births, of which 13 988 infants were alive at 1 year of age. For our analyses, we used data from 11 649 mother-child pairs who had data on AD from at least 1 survey and sleep outcomes from at least 1 survey through age 11 years (83.3% of children who remained alive at 1 year of age). The study website contains details of all the data available through a fully searchable data dictionary and variable search tool.^18^ Data collection began in 1990 through 1992, and analyses for this study, a secondary analysis of this cohort, were performed from September 2017 through September 2018. Ethical approval for the study was obtained from the ALSPAC Ethics and Law Committee and the Local Research Ethics Committees. The University of California, San Francisco declared the study exempt from the need for institutional review board approval because all data obtained by investigators were fully deidentified. Participants provided written informed consent at enrollment into the original cohort.

### Exposure

The primary exposure was a time-varying measure of active AD in the child. Mothers were asked a standardized question about flexural dermatitis at 10 time points between child ages 6 months and 11 years (6, 18, 30, 42, 57, 69, 81, 103, 128, and 140 months): “Has your child had an itchy, dry skin rash in the joints and creases of his body (eg, behind the knees, elbows, under the arms) in the past year?” This question is comparable to that used in the large International Study of Asthma and Allergies in Childhood (ISAAC).^19^ Children were categorized as having active AD if their mother reported flexural dermatitis at least twice, up to and including the time point being considered.^20,21,22^ Disease severity was assessed at each time point by asking mothers to categorize their child’s flexural dermatitis over the past year as no problem, mild, moderate, or severe.

### Maternal Sleep Outcomes

Five maternal sleep outcomes were measured at various time points throughout childhood (Table 1). Sleep duration was measured by a standardized categorical question repeated at 5 time points: *“*How many hours of sleep do you get altogether now during an average night?” (Answer choices: 1-3 hours, 4-5 hours, 6-7 hours, and >7 hours). This variable was modeled as a dichotomous outcome—fewer than 6 hours per night compared with 6 or more hours per night—because the National Sleep Foundation recommends 7 to 9 hours of sleep for adults aged 26 to 64 years.^23^ Difficulty falling asleep was assessed at 3 time points by the question, “Can you go to sleep alright?” (Answer choices: never/not very often vs very often/often), and early morning awakening was assessed at the same 3 time points by the question, “Do you wake unusually early in the morning even when you haven’t been woken by your child or family?” (Answer choices: very often/often vs never/not very often). Subjectively insufficient sleep was examined at 4 time points by the question, “Do you feel that you are getting enough sleep?” (Answer choices: yes/no). Finally, daytime exhaustion was measured at 7 time points by the question, “In the past month, how often have you felt exhausted?” (Answer choices: almost always vs sometimes/not at all.)

**Table 1.** Maternal Sleep Outcome Measurements by Child Age

Outcome	Child Age, mo^a^							
	21	33	47	61	73	85	110	134
Sleep duration	x	x		x		x		x
Early morning awakening	x	x		x				
Difficulty falling asleep	x	x		x				
Subjectively insufficient sleep	x	x		x		x		
Daytime exhaustion	x	x	x	x	x		x	x

^a^ An *x* indicates that the outcome was reported at the time point.

### Additional Covariates

Several potential confounders, mediators, and effect modifiers were identified from earlier literature and incorporated into a directed acyclic graph that was used to guide our modeling strategy (eFigure in the Supplement). These factors included child and mother demographic characteristics (child sex, child age, mother’s race/ethnicity, and maternal age at delivery), indicators of socioeconomic status, household smoking exposure, presence of comorbid atopic diseases in the child (asthma or allergic rhinitis), maternal sleep problems during pregnancy, maternal history of any atopic condition (AD, asthma, or allergic rhinitis), and child sleep disturbances. Socioeconomic status was measured using prenatal questionnaires collected from parents at study enrollment, including the mother’s highest educational attainment, social class based on occupation (highest of either parent), household crowding index (number of people living in the household divided by the number of rooms in the house), and a financial difficulties score assessing the mother’s self-reported difficulty to afford food, clothing, heating, rent or mortgage, and items necessary to care for her baby. Maternal sleep problems during pregnancy were assessed by prenatal questionnaire and based on questions about difficulty falling asleep at 18 and 32 weeks’ gestation.

Time-varying covariates included child comorbid atopic disease, household smoking exposure, and child sleep disturbances. A child was determined to have comorbid atopic disease at each time point examined if his or her mother reported asthma symptoms, allergic rhinitis symptoms, or both at that time, on questions similar to those used in the ISAAC studies.^19^ Models also included a measure of household smoking exposure, which was assessed by maternal questionnaire about the number of smokers living in the household at multiple time points throughout childhood. Finally, child sleep disturbances were measured by a standardized question about the frequency of nighttime awakenings (dichotomized at ≥1 awakening per night) at 6 time points in early childhood (30, 42, 57, 69, 81, and 115 months). Child nighttime awakenings have been shown to be a valid measure of AD-associated child sleep disturbances throughout childhood.^24^

### Missing Data

As has been described in detail elsewhere, there are both intermittent missing data and attrition (ie, loss to follow-up) in the ALSPAC cohort.^16^ Multiple imputation was used to impute missing exposure, outcome, and covariate data. Iterative chained equations were used because most variables in our models did not follow a normal distribution. Fifty imputed data sets were generated and used to repeat primary analyses.

### Statistical Analysis

Cross-sectional logistic regression analyses were performed to compare maternal sleep outcomes between mothers of children with and without AD at each time point. Longitudinal analyses with repeated measures of the exposure, outcome, and time-varying covariates were then performed using mixed-effects models with random slopes and intercepts for each individual to estimate the participant-specific odds ratios (ORs) for each of the maternal sleep outcomes. The minimally sufficient adjustment set was determined using our directed acyclic graph (eFigure in the Supplement), and these factors were included in multivariable models. Multivariable model 1 represents the fully adjusted model used to calculate the total association of child AD with maternal sleep, and model 2 was additionally adjusted for child sleep disturbances at each time point to estimate the direct association of child AD with maternal sleep, independent of child sleep disturbances. Interactions between child AD and child comorbid atopic disease (asthma or allergic rhinitis), child age, child sex, and maternal educational attainment were tested. All statistical analyses were performed using Stata, version 14.2 (StataCorp). Statistical significance was set at *P* < .05.

## Results

The study sample was composed of 13 988 mother-child pairs. Among the children, 7220 of 13 978 (51.7%) were male. Of the mothers, 12 001 of 12 324 (97.4%) were of white race/ethnicity, and 11 585 of 13 972 (82.9%) were aged 21 to 34 years. The sample was followed for a median duration of 11 years (interquartile range, 7-11 years). Mothers of children with AD were more likely to have a personal history of atopy and a higher educational attainment and social class (Table 2). The annual period prevalence of child AD ranged from 16.4% to 21.0%, and 21.8% to 38.5% of children with AD were reported to have moderate to severe disease at any given time point. Overall, 4767 children of 13 988 (34.1%) met the definition of having AD between 2 and 11 years of age.

**Table 2.** Cohort Characteristics

Characteristic	No./Total No. (%)	Dermatitis, No. (%)		*P* Value^**b**^
		No AD (n = 5599)	AD (n = 4767)^a^	
**Maternal Characteristics**				
White race/ethnicity	12 001/12 324 (97.4)	5039 (97.7)	4500 (97.9)	.51
Age at delivery, y				
≤20	1004/13 972 (7.2)	376 (6.7)	202 (4.2)	<.001
21-34	11 585/13 972 (82.9)	4630 (82.7)	4061 (85.2)	
≥35	1383/13 972 (9.9)	593 (10.6)	504 (10.6)	
Maternal history of atopic condition^c^	5659/12 454 (45.4)	2179 (42.0)	2318 (50.3)	<.001
Household smoking exposure^d^	3683/10 188 (36.2)	1661 (37.4)	1435 (33.7)	<.001
Maternal highest educational attainment^e^				
CSE/none	2504/12 412 (20.2)	1106 (21.3)	701 (15.2)	<.001
Vocational	1224/12 412 (9.9)	513 (9.9)	435 (9.4)	
O level	4294/12 412 (34.6)	1860 (35.8)	1595 (34.6)	
A level	2791/12 412 (22.5)	1149 (22.1)	1141 (24.8)	
Degree	1599/12 412 (12.9)	563 (10.9)	736 (16.0)	
Social class^f^				
Unskilled	227/12 254 (1.9)	87 (1.7)	53 (1.2)	<.001
Partly skilled	920/12 254 (7.5)	381 (7.5)	274 (6.2)	
Skilled manual	1666/12 254 (13.6)	717 (14.2)	502 (11.3)	
Skilled nonmanual	3780/12 254 (30.8)	1601 (31.7)	1340 (30.2)	
Managerial and technical	4566/12 254 (37.3)	1878 (37.1)	1779 (40.0)	
Professional	1095/12 254 (8.9)	395 (7.8)	493 (11.1)	
Financial difficulties quartile^g^				
Lowest	4337/12 083 (35.9)	1819 (36.0)	1722 (38.5)	.06
Mild	3006/12 083 (24.9)	1272 (25.1)	1104 (24.7)	
Moderate	2324/12 083 (19.2)	973 (19.2)	832 (18.6)	
Highest	2416/12 083 (20.0)	995 (19.7)	817 (18.3)	
Crowding index^h^				
≤0.5	5329/12 799 (41.6)	2085 (40.0)	2146 (46.7)	<.001
>0.5-0.75	4013/12 799 (31.3)	1644 (31.6)	1456 (31.7)	
>0.75-1.0	2579/12 799 (20.2)	1111 (21.3)	788 (17.2)	
>1.0	878/12 799 (6.9)	370 (7.1)	203 (4.4)	
Maternal sleep problems during pregnancy^i^	4535/13 406 (33.8)	1886 (34.6)	1486 (31.5)	.001
**Child Characteristics**				
Male	7220/13 978 (51.7)	3022 (54.0)	2319 (48.7)	<.001
White race/ethnicity	11 468/12 077 (95.0)	4812 (95.5)	4335 (95.6)	.82
Asthma ever^j^	3237/12 612 (25.7)	1078 (19.3)	1745 (36.6)	<.001
Allergic rhinitis ever^i^	1375/10 156 (13.5)	356 (8.1)	872 (20.2)	<.001
Asthma or allergic rhinitis ever^i^	3919/12 620 (31.1)	1300 (23.2)	2120 (44.5)	<.001
Asthma and allergic rhinitis ever^i^	693/12 620 (5.5)	134 (2.4)	497 (10.4)	<.001

Abbreviations: AD, atopic dermatitis; CSE, certificate of secondary education.

^a^ Children who ever reported AD through age 11 years (ie, ≥2 reports of flexural dermatitis). There were 3622 additional children with only 1 report of flexural dermatitis.

^b^ The χ^2^ test comparing mother-child pairs who never reported AD with those who ever reported AD through age 11 years.

^c^ Includes AD, asthma, or allergic rhinitis.

^d^ When child was aged 1.75 years.

^e^ United Kingdom education levels: CSE (national school examinations at 16 years); vocational; O level (national school examinations at 16 years, higher than CSE); A level (national school examinations at 18 years); and degree (university degree or higher).

^f^ Higher of either parent based on occupation.

^g^ Financial difficulties score assessed the mother’s self-reported difficulty to afford food, clothing, heating, rent or mortgage, and items necessary to care for her baby. The total score was divided into quartiles, increasing with greater financial difficulties.

^h^ Defined as the number of persons living in the household divided by the number of rooms in the household.

^i^ Defined as difficulty falling asleep often or very often at 18 or 32 weeks’ gestation.

^j^ Defined as at least 2 reports of symptoms throughout childhood.

The proportion of all mothers who reported each of the 5 sleep outcomes was fairly consistent across ages: 5% to 12% reported sleeping less than 6 hours per night, 18% to 20% reported early morning awakenings, 12% to 13% reported difficulty falling asleep, 38% to 43% reported subjectively insufficient sleep, and 6% to 10% reported daytime exhaustion during the first 11 years of their child’s life.

In unadjusted cross-sectional analyses comparing mothers of children with and without AD at individual time points, results were generally consistent across all child ages (eTable 1 in the Supplement). In longitudinal analyses adjusted for confounders, sleep duration (adjusted OR [AOR], 1.09; 95% CI, 0.90-1.32) and early morning awakening (AOR, 1.16; 95% CI, 0.93-1.46) were similar for mothers of children with active AD compared with mothers of children who never reported AD (Table 3). In contrast, mothers of children with active AD were more likely to report difficulty falling asleep (AOR, 1.36; 95% CI, 1.01-1.83), subjectively insufficient sleep (AOR, 1.43; 95% CI, 1.24-1.66), and daytime exhaustion (AOR, 1.41; 95% CI, 1.12-1.78) (Table 3). None of the interactions between child AD and child comorbid atopic disease (asthma or allergic rhinitis), child age, child sex, and maternal educational attainment were statistically significant.

**Table 3.** Odds of Maternal Sleep Disturbances Across Time Points Among Mothers of Children With Active Atopic Dermatitis (AD) Compared With Mothers of Children Who Never Reported AD^a^

Model	Odds Ratio (95% CI)				
	Sleep Duration <6 h per Night^b^	Early Morning Awakening^c^	Difficulty Falling Asleep^d^	Subjectively Insufficient Sleep^e^	Daytime Exhaustion^f^
Unadjusted	0.92 (0.78-1.09)	1.27 (1.06-1.52)	1.17 (0.96-1.43)	1.45 (1.27-1.66)	1.38 (1.13-1.69)
Model 1	1.09 (0.90-1.32)	1.16 (0.93-1.46)	1.36 (1.01-1.83)	1.43 (1.24-1.66)	1.41 (1.12-1.78)
Model 2	1.06 (0.85-1.33)	1.15 (0.91-1.44)	1.33 (0.99-1.79)	1.42 (1.23-1.65)	1.43 (1.12-1.83)

^a^ For each of the maternal sleep outcomes, results from an unadjusted and 2 separate adjusted multivariable mixed models examining the association between child active AD and maternal sleep disturbances at multiple time points are shown. Model 1 adjusted for child sex, child age, mother’s race/ethnicity, child atopy (asthma and/or allergic rhinitis), household smoking exposure, maternal educational attainment, social class, crowding index, financial difficulties score, maternal sleep problems during pregnancy, maternal atopy, and maternal age at delivery. Model 2 adjusted for the same variables as model 1 as well as child sleep disturbances at each time point. Model 2 excluded the 134-month time point from analyses for the outcomes of sleep duration and daytime exhaustion because data on child sleep disturbances were not available at that time.

^b^ Unadjusted model: 10 691 individuals; mean (range) observations per individual, 3.7 (1-5). Model 1: 8961 individuals; mean (range) observations per individual, 3.7(1-5). Model 2: 8837 individuals; mean (range) observations per individual, 3.1 (1-4).

^c^ Unadjusted model: 10 449 individuals; mean (range) observations per individual, 2.5 (1-3). Model 1: 8796 individuals; mean (range) observations per individual, 2.5 (1-3). Model 2: 8771 individuals; mean (range) observations per individual, 2.4 (1-3).

^d^ Unadjusted model: 10 443 individuals; mean (range) observations per individual, 2.5 (1-3). Model 1: 8794 individuals; mean (range) observations per individual, 2.5 (1-3). Model 2: 8768 individuals; mean (range) observations per individual, 2.4 (1-3).

^e^ Unadjusted model: 10 546 individuals; mean (range) observations per individual, 3.1 (1-4). Model 1: 8883 individuals; mean (range) observations per individual, 3.1 (1-4). Model 2: 8831 individuals; mean (range) observations per individual, 3.0 (1-4).

^f^ Unadjusted model: 10 886 individuals; mean (range) observations per individual, 5.1 (1-7). Model 1: 9061 individuals; mean (range) observations per individual, 5.0 (1-7). Model 2: 9003 individuals; mean (range) observations per individual, 4.4 (1-6).

### Subanalysis With Child AD Disease Severity

When we examined the odds of maternal sleep disturbances according to the child’s disease severity, we found larger effects among mothers of children with more severe disease (Figure and eTable 2 in the Supplement). For maternal sleep duration, we only found a statistically significant association for mothers of children with severe disease, who had 61% greater odds of reporting sleep duration less than 6 hours per night (AOR, 1.61; 95% CI, 1.05-2.48). Although the odds were also greater for early morning awakening and difficulty falling asleep, these odds did not reach statistical significance (early awakening: AOR, 1.53; 95% CI, 0.96-2.45; difficulty falling asleep: AOR, 1.53; 95% CI, 0.90-2.58). An association with daytime exhaustion and subjectively insufficient sleep was stronger for mothers of children with severe disease (72%-89% greater odds; subjectively insufficient sleep, OR, 1.89; 95% CI, 1.34-2.66; daytime exhaustion, OR, 1.72; 95% CI, 1.12-2.64) and remained significant for those with mild to moderate disease (31%-39% greater odds; moderate disease: subjectively insufficient sleep, OR, 1.31; 95% CI, 1.09-1.57; daytime exhaustion, OR, 1.38; 95% CI, 1.06-1.81; mild disease: subjectively insufficient sleep, OR, 1.34; 95% CI, 1.17-1.54; daytime exhaustion, OR, 1.39; 95% CI, 1.12-1.74).

**Figure.**
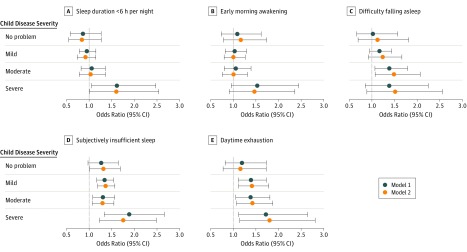
Adjusted Odds for Sleep Disturbances Across Time Points Among Mothers of Children With Active Atopic Dermatitis (AD) by Disease Severity vs Mothers of Children Who Never Reported AD For each of the 5 maternal sleep outcomes, results from 2 separate, adjusted, multivariable mixed models examining the association between child active AD and maternal sleep disturbances at multiple time points are shown. Model 1 is adjusted for child sex, child age, mother’s race/ethnicity, child atopy (asthma and/or allergic rhinitis), household smoking exposure, maternal educational attainment, social class, crowding index, financial difficulties score, maternal sleep problems during pregnancy, maternal atopy, and maternal age at delivery. Model 2 is adjusted for the same variables as model 1 as well as child sleep disturbances at each time point. Model 2 excluded the 134-month time point from analyses of the outcomes of sleep duration and daytime exhaustion because data on child sleep disturbances were not available at that time.

### Subanalysis Accounting for Child Sleep Disturbances

Finally, we recalculated the odds of maternal sleep disturbances while controlling for child sleep disturbances at each time point to examine whether child sleep accounted for most of the association between child AD and maternal sleep. Overall, we found that the magnitude and significance of the associations was largely unchanged after adjusting for child sleep disturbances in model 2 (Table 3, Figure, and eTable 2 in the Supplement).

### Multiple Imputation Results

Imputed data sets were used to repeat all primary analyses (eTables 2 and 3 in the Supplement). Estimates using the imputed data were slightly attenuated toward the null; however, results remained qualitatively similar and largely consistent with those from the primary data.

## Discussion

In a cohort of 11 649 mother-child pairs followed up from the child’s birth through age 11 years, sleep duration was similar between mothers of children with and without AD. However, mothers of children with active AD reported subjectively insufficient sleep, difficulty falling asleep, and daytime exhaustion throughout the first 11 years of childhood. These findings have important implications given that AD is one of the most common chronic childhood conditions and affects up to 20% of children of any age in industrialized countries.^25^

A strength of the present study is the use of a large prospective, longitudinal, population-based cohort in which children were followed up from birth for more than 10 years. The few studies that have specifically examined the association of child AD with parental sleep relative to parents of children without AD have been small, cross-sectional, and conducted in clinic-based populations.^12,13,14,15,26^ In our study, only mothers of children with severe disease were significantly more likely to report sleeping less than 6 hours per night. Similarly, Moore and colleagues^26^ found that parents of children with moderate to severe AD lost a median of 39 minutes of sleep per night, and Meltzer and Booster^13^ found that parents of children with AD were significantly more likely to report sleep duration of less than 6 hours per night compared with parents of healthy children.

Although impaired parental sleep is thought to be related to the child’s nighttime awakenings,^12,13,26^ adjusting for child sleep disturbances in our study did not change our conclusions. This finding suggests that maternal sleep disturbances may be explained by other factors unrelated to the child’s sleep disturbances. Recent work from the pediatric psychology literature in the setting of other chronic illnesses, including asthma, ventilator dependency, and cystic fibrosis, has supported caregiver stress and anxiety related to the child’s condition as potential contributors to parental sleep disruptions.^7,8,13,27,28^ Other studies have found that parents of children with AD experience high rates of psychological distress and depression,^12,29,30,31^ which may explain some of their sleep disturbances. Further research is needed to better understand the exact causes of these sleep disruptions and to characterize their consequences on daytime functioning, well-being, and health outcomes in caregivers of children with AD.

Maternal emotional and psychosocial well-being is inextricably linked to the child’s health, development, and cognitive and social functioning.^1,2,32^ In children with AD, chronically sleep-deprived, exhausted, or depressed caregivers may be less equipped to implement time-consuming treatment regimens, regulate their child’s behavior, and help the child cope with his or her illness.^13,33^ The American Academy of Pediatrics recommends that clinicians provide family-oriented care that addresses the needs of the entire family and promotes family functioning to optimize child outcomes.^1^ These recommendations include screening for family circumstances that may be associated with unfavorable outcomes for the well-being of the child. Our results suggest that clinicians caring for children with AD should screen for caregiver sleep disturbances and fatigue, engage mothers in discussion about their emotional health, and consider offering resources for psychosocial support, particularly for mothers of children with more severe disease. Clinicians may also consider formally screening mothers for sleep problems using the Pittsburgh Sleep Quality Index,^34^ for depression using the Patient Health Questionnaire-9,^35,36^ and for anxiety using the Generalized Anxiety Disorder 7-item scale,^37^ all of which are well-validated questionnaires. Additional research is needed to understand how interventions targeted to both child AD and maternal wellness can improve the entire family’s sleep.

### Limitations

Several limitations warrant discussion, including the potential for selection bias due to missing data and attrition, which is inherent to all large-scale longitudinal studies. To address this limitation, we performed multiple imputations and, reassuringly, found similar results. Another important limitation relates to the possibility for misclassification bias because both exposure and outcome data were self-reported. Although we cannot rule out the possibility of some misclassification, previous studies have found that self-report of AD closely approximates physician assessment.^38^ Although it is possible that mothers who are chronically exhausted may have a tendency to report more severe disease in their child, the associations remained significant for mothers of children with mild disease. In addition, the estimates of moderate to severe disease match those of other population-based studies,^24,39^ and parent-reported disease severity has been shown to correlate well with objective disease severity measures.^40,41^ In population-based studies comparing survey responses about sleep duration with objective measures, self-report tends to overestimate total duration of sleep,^42^ which would bias our results toward the null. An additional limitation is that the sleep quality questions about difficulty falling asleep and early morning awakenings were not asked beyond the child’s fifth year. Future work should investigate whether these maternal sleep quality disturbances persist into later childhood and adolescence. The present study focused on mothers’ sleep because mothers are often the primary caregivers; future research should consider the role of fathers and other caregivers. Finally, although the cohort is fairly representative of the United Kingdom’s population,^16^ the extent to which our results are generalizable to other populations is unclear.

## Conclusions

This study’s results found that, throughout the first 11 years of childhood, mothers of children with active AD experienced more difficulty falling asleep, subjectively insufficient sleep, and daytime exhaustion compared with mothers of children without AD, and the child’s disease severity was associated with worse maternal sleep. Child sleep disturbances did not fully explain maternal sleep disturbances, and further research is needed to better understand these mechanisms. In caring for children with AD, clinicians should inquire about caregiver sleep disturbances and fatigue and consider offering psychosocial support.

## Notes

Supplement. **eFigure.** Directed Acyclic Graph **eTable 1**. Unadjusted Odds of Sleep Disturbances Among Mothers of Children With Active Atopic Dermatitis by Child Age Compared to Mothers of Children Who Never Reported Atopic Dermatitis **eTable 2.** Odds and 95% Confidence Intervals for Maternal Sleep Disturbances Across Time Points Among Mothers of Children With Active Atopic Dermatitis According to Disease Severity Compared to Mothers of Children Who Never Reported Atopic Dermatitis (Non-Imputed Data) **eTable 3.** Odds and 95% Confidence Intervals for Maternal Sleep Disturbances Across Time Points Among Mothers of Children With Active Atopic Dermatitis According to Disease Severity Compared to Mothers of Children Who Never Reported Atopic Dermatitis (Imputed Data)
