# Supplementary material for: Assessment of Sleep Disturbances and Exhaustion in Mothers of Children With Atopic Dermatitis
Source: JAMA Dermatol. 2019 Mar 20;155(5):556–63. doi: 10.1001/jamadermatol.2018.5641 (PMC6506883; doi:10.1001/jamadermatol.2018.5641)
Supplement: Supplement. — eFigure. Directed Acyclic Graph eTable 1. Unadjusted Odds of Sleep Disturbances Among Mothers of Children With Active Atopic Dermatitis by Child Age Compared to Mothers of Children Who Never Reported Atopic Dermatitis eTable 2. Odds and 95% Confidence Intervals for Maternal Sleep Disturbances Across Time Points Among Mothers of Children With Active Atopic Dermatitis According to Disease Severity Compared to Mothers of Children Who Never Reported Atopic Dermatitis (Non-Imputed Data) eTable 3. Odds and 95% Confidence Intervals for Maternal Sleep Disturbances Across Time Points Among Mothers of Children With Active Atopic Dermatitis According to Disease Severity Compared to Mothers of Children Who Never Reported Atopic Dermatitis (Imputed Data) [file jamadermatol-155-556-s001.pdf]

Supplementary Online Content

Ramirez FD, Chen S, Langan SM, et al. Assessment of sleep disturbances and exhaustion in mothers of children with atopic dermatitis. *JAMA Dermatol*. Published online March 20, 2019. doi:10.1001/jamadermatol.2018.5641

eFigure. Directed Acyclic Graph

eTable 1. Unadjusted Odds of Sleep Disturbances Among Mothers of Children With Active Atopic Dermatitis by Child Age Compared to Mothers of Children Who Never Reported Atopic Dermatitis

eTable 2. Odds and 95% Confidence Intervals for Maternal Sleep Disturbances Across Time Points Among Mothers of Children With Active Atopic Dermatitis According to Disease Severity Compared to Mothers of Children Who Never Reported Atopic Dermatitis (Non-Imputed Data)

eTable 3. Odds and 95% Confidence Intervals for Maternal Sleep Disturbances Across Time Points Among Mothers of Children With Active Atopic Dermatitis According to Disease Severity Compared to Mothers of Children Who Never Reported Atopic Dermatitis (Imputed Data)

This supplementary material has been provided by the authors to give readers additional information about their work.

© 2019 Ramirez BA et al. *JAMA Dermatology*.

eFigure. Directed Acyclic Graph

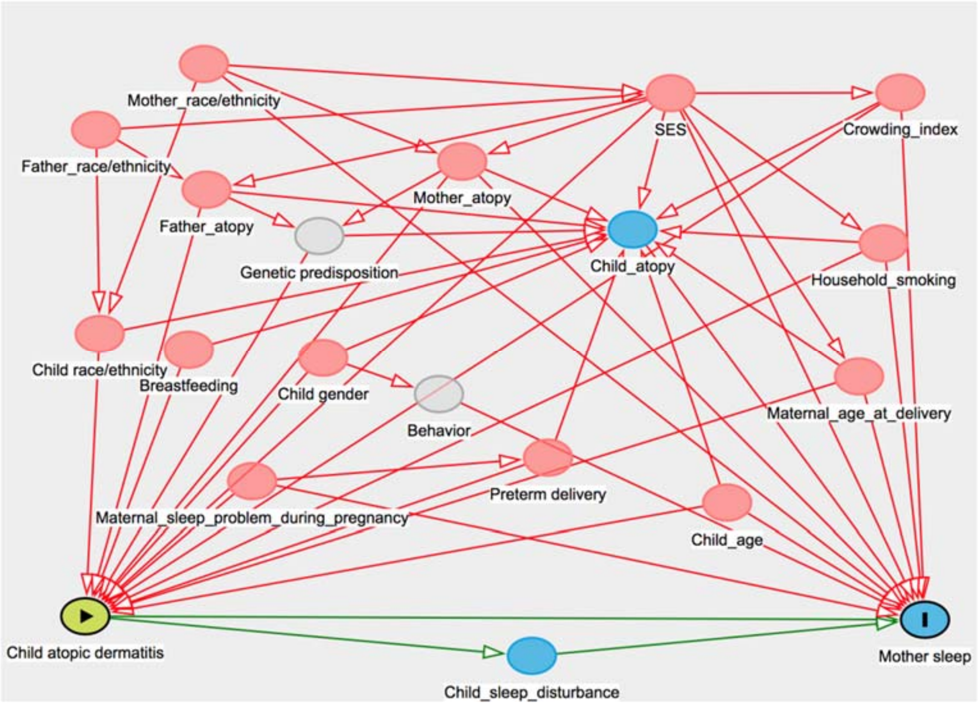

eFigure. Directed acyclic graph representing relationships between covariates and primary predictor and outcome. Red circles represent ancestors of the exposure and outcome (i.e. confounders), blue circles represent ancestors of the outcome (i.e. causal determinants of the outcome), and grey circles represent unobserved (i.e. latent) variables. Green lines represent causal paths, and red lines represent biasing paths. The minimally sufficient adjustment set (MSAS) was determined using DAGitty, a software for creating causal diagrams to minimize confounding bias in epidemiology.[34] Child atopy (asthma or allergic rhinitis) was considered to be a collider which was appropriately accounted for by adjusting for additional variables contained on the backdoor paths shared by this collider. The final MSAS was determined to be the following: child gender, child age, mother race/ethnicity, maternal age at delivery, social class, maternal education, financial difficulties score, crowding index, presence of comorbid atopic diseases in the child (asthma or allergic rhinitis), maternal history of any atopic condition (atopic dermatitis, asthma, or allergic rhinitis), household smoking exposure, and maternal sleep problems during pregnancy.

© 2019 Ramirez BA et al. *JAMA Dermatology*.

eTable 1. Unadjusted Odds of Sleep Disturbances Among Mothers of Children With Active Atopic Dermatitis by Child Age Compared to Mothers of Children Who Never Reported Atopic Dermatitis

Child age	Sleep duration < 6 hours per night	Early morning awakening	Difficulty falling asleep	Subjectively insufficient sleep	Daytime exhaustion
	Odds Ratio (95% Confidence Interval)				
21 months	0.96 (0.78 - 1.18)	**1.20 (1.01 - 1.42)**	1.09 (0.90 - 1.33)	**1.37 (1.20 - 1.57)**	**1.55 (1.26 - 1.90)**
33 months	1.14 (0.92 - 1.42)	1.09 (0.91 - 1.30)	1.11 (0.89 - 1.38)	**1.43 (1.24 - 1.65)**	**1.33 (1.06 - 1.68)**
47 months	-	-	-	-	**1.37 (1.06 - 1.78)**
61 months	1.06 (0.82 - 1.38)	1.17 (0.96 - 1.43)	1.17 (0.91 - 1.50)	**1.29 (1.09 - 1.52)**	**1.79 (1.31 - 2.44)**
73 months	-	-	-	-	0.97 (0.72 - 1.31)
85 months	1.15 (0.86 - 1.55)	-	-	**1.38 (1.15 - 1.65)**	-
110 months	-	-	-	-	1.28 (0.82 - 2.00)
134 months	1.02 (0.62 - 1.69)	-	-	-	**1.60 (1.12 - 2.27)**

eTable 1. For each of the maternal sleep outcomes, results from unadjusted logistic regression models examining the association between child active atopic dermatitis and maternal sleep outcomes based on cross-sectional data at different child ages.

© 2019 Ramirez BA et al. *JAMA Dermatology*.

eTable 2. Odds and 95% Confidence Intervals for Maternal Sleep Disturbances Across Time Points Among Mothers of Children With Active Atopic Dermatitis According to Disease Severity Compared to Mothers of Children Who Never Reported Atopic Dermatitis (Non-Imputed Data)

	**Child atopic dermatitis disease activity and severity**	**Sleep duration < 6 hours***	**Early morning awakening**	**Difficulty falling asleep**	**Subjectively insufficient sleep**	**Daytime exhaustion***
	Never reported atopic dermatitis	Ref.	Ref.	Ref.	Ref.	Ref.
Unadjusted	No problem	**0.68 (0.48 - 0.95)**	1.18 (0.83 - 1.66)	0.91 (0.61 - 1.36)	**1.28 (1.01 - 1.62)**	1.10 (0.78 - 1.53)
	Mild	**0.70 (0.59 - 0.84)**	1.07 (0.89 - 1.28)	1.02 (0.83 - 1.24)	**1.33 (1.17 - 1.51)**	**1.22 (1.01 - 1.48)**
	Moderate	1.02 (0.81 - 1.28)	1.16 (0.92 - 1.47)	**1.31 (1.02 - 1.68)**	**1.36 (1.15 - 1.61)**	**1.42 (1.11 - 1.80)**
	Severe	**1.49 (1.02 - 2.16)**	**1.79 (1.20 - 2.67)**	1.55 (1.00 - 2.40)	**1.92 (1.42 - 2.61)**	**1.92 (1.32 - 2.79)**
Model 1	No problem	0.86 (0.59 - 1.27)	1.09 (0.73 - 1.62)	1.13 (0.70 - 1.82)	1.27 (0.98 - 1.65)	1.19 (0.82 - 1.73)
	Mild	0.95 (0.79 - 1.15)	1.03 (0.82 - 1.30)	1.29 (0.96 - 1.73)	**1.34 (1.17 - 1.54)**	**1.39 (1.12 - 1.74)**
	Moderate	1.06 (0.82 - 1.36)	1.06 (0.80 - 1.39)	**1.53 (1.10 - 2.12)**	**1.31 (1.09 - 1.57)**	**1.38 (1.06 - 1.81)**
	Severe	**1.61 (1.05 - 2.48)**	1.53 (0.96 - 2.45)	1.53 (0.90 - 2.58)	**1.89 (1.34 - 2.66)**	**1.72 (1.12 - 2.64)**
Model 2	No problem	0.84 (0.55 - 1.29)	1.17 (0.79 - 1.74)	1.12 (0.70 - 1.81)	**1.32 (1.01 - 1.71)**	1.16 (0.78 - 1.73)
	Mild	0.92 (0.74 - 1.15)	1.01 (0.80 - 1.26)	1.24 (0.92 - 1.66)	**1.37 (1.19 - 1.57)**	**1.42 (1.12 - 1.79)**
	Moderate	1.03 (0.78 - 1.36)	1.01 (0.76 - 1.33)	**1.49 (1.07 - 2.07)**	**1.30 (1.08 - 1.56)**	**1.43 (1.07 - 1.89)**
	Severe	**1.60 (1.01 - 2.54)**	1.47 (0.91 - 2.36)	1.51 (0.89 - 2.56)	**1.76 (1.23 - 2.50)**	**1.80 (1.15 - 2.82)**

eTable 2. For each of the maternal sleep outcomes, results from an unadjusted and two separate adjusted multivariable mixed models examining the association between child atopic dermatitis disease severity and maternal sleep disturbances at multiple time points. Model 1 adjusted for child gender, child age, mother race/ethnicity, child atopy (asthma and/or allergic rhinitis), household smoking exposure, maternal education, social class, crowding index, financial difficulties score, maternal sleep problems during pregnancy, maternal atopy, and maternal age at delivery. Model 2 adjusted for the same variables as Model 1, as well as child sleep disturbances at each time point. * Model 2 excluded the 134-month time point from analyses for the outcomes of sleep duration and daytime exhaustion as data on child sleep disturbances was not available at this time point. Results using non-imputed original data.

© 2019 Ramirez BA et al. *JAMA Dermatology*.

eTable 3. Odds and 95% Confidence Intervals for Maternal Sleep Disturbances Across Time Points Among Mothers of Children With Active Atopic Dermatitis According to Disease Severity Compared to Mothers of Children Who Never Reported Atopic Dermatitis (Imputed Data)

	**Child atopic dermatitis disease activity and severity**	**Sleep duration < 6 hours***	**Early morning awakening**	**Difficulty falling asleep**	**Subjectively insufficient sleep**	**Daytime exhaustion***
	Never reported atopic dermatitis	Ref.	Ref.	Ref.	Ref.	Ref.
Unadjusted	No problem	0.89 (0.71 - 1.11)	1.12 (0.92 - 1.36)	1.00 (0.79 - 1.28)	**1.20 (1.04 - 1.37)**	1.19 (0.97 - 1.45)
	Mild	0.90 (0.81 - 1.01)	1.08 (0.97 - 1.20)	1.11 (0.98 - 1.25)	**1.24 (1.14 - 1.34)**	**1.31 (1.18 - 1.45)**
	Moderate	1.08 (0.91 - 1.27)	1.13 (0.98 - 1.30)	**1.23 (1.05 - 1.45)**	**1.27 (1.14 - 1.40)**	**1.43 (1.22 - 1.67)**
	Severe	1.28 (1.00 - 1.65)	**1.36 (1.06 - 1.75)**	**1.34 (1.01 - 1.78)**	**1.49 (1.26 - 1.76)**	**1.64 (1.28 - 2.08)**
Model 1	No problem	1.01 (0.81 - 1.27)	1.13 (0.92 - 1.39)	1.06 (0.82 - 1.38)	**1.21 (1.06 - 1.39)**	1.22 (0.99 - 1.50)
	Mild	1.01 (0.90 - 1.13)	1.07 (0.96 - 1.19)	**1.14 (1.00 - 1.30)**	**1.24 (1.14 - 1.35)**	**1.34 (1.21 - 1.49)**
	Moderate	1.11 (0.94 - 1.32)	1.11 (0.95 - 1.28)	**1.25 (1.05 - 1.48)**	**1.24 (1.11 - 1.37)**	**1.43 (1.21 - 1.67)**
	Severe	1.28 (0.99 - 1.65)	**1.31 (1.01 - 1.68)**	1.29 (0.96 - 1.73)	**1.44 (1.21 - 1.72)**	**1.60 (1.25 - 2.04)**
Model 2	No problem	1.00 (0.79 - 1.28)	1.13 (0.91 - 1.39)	1.06 (0.82 - 1.37)	**1.21 (1.05 - 1.39)**	1.17 (0.94 - 1.45)
	Mild	0.99 (0.87 - 1.12)	1.07 (0.96 - 1.19)	**1.14 (1.00 - 1.30)**	**1.24 (1.14 - 1.34)**	**1.32 (1.18 - 1.48)**
	Moderate	1.10 (0.93 - 1.31)	1.10 (0.95 - 1.28)	**1.24 (1.05 - 1.47)**	**1.22 (1.10 - 1.36)**	**1.39 (1.19 - 1.64)**
	Severe	1.27 (0.99 - 1.63)	**1.29 (1.00 - 1.66)**	1.28 (0.95 - 1.71)	**1.41 (1.19 - 1.68)**	**1.57 (1.23 - 2.02)**

eTable 3. For each of the maternal sleep outcomes, results from an unadjusted and two separate adjusted multivariable generalized estimating equation (GEE) models examining the association between child atopic dermatitis disease severity and maternal sleep disturbances at multiple time points. Model 1 adjusted for child gender, child age, mother race/ethnicity, child atopy (asthma and/or allergic rhinitis), household smoking exposure, maternal education, social class, crowding index, financial difficulties score, maternal sleep problems during pregnancy, maternal atopy, and maternal age at delivery. Model 2 adjusted for the same variables as Model 1, as well as child sleep disturbances at each time point. * Model 2 excluded the 134-month time point from analyses for the outcomes of sleep duration and daytime exhaustion as data on child sleep disturbances was not available at this time point. Results using imputed data. Due to the extremely large size of the imputed dataset, mixed models were not computationally feasible and GEE models were used instead. Robust standard errors were used in the GEE models.

© 2019 Ramirez BA et al. *JAMA Dermatology*.
